# Porcine Urinary Bladder Matrix for Management of Infected Radiation Mastectomy Wound

**DOI:** 10.7759/cureus.1451

**Published:** 2017-07-10

**Authors:** Yana Puckett, Theophilus Pham, Shirley McReynolds, Catherine A Ronaghan

**Affiliations:** 1 Department of Surgery, Texas Tech University Health Sciences Center; 2 School of Medicine, Texas Tech University Health Sciences Center

**Keywords:** non-healing mastectomy wound, acell, porcine urinary bladder matrix

## Abstract

We present a case report on the successful healing of a Pseudomonas infection wound in a 52-year-old female with morbid obesity, noninsulin dependent diabetes mellitus and a history of tobacco use, who presented with Stage IIIA (T3, N2, Mo) infiltrating ductal carcinoma. The patient received neoadjuvant chemotherapy prior to her bilateral skin-sparing total mastectomies with right axillary sentinel lymphadenectomy. She also had staged reconstruction with temporary breast implants and plans for deep inferior epigastric perforator flaps. Two months after chest wall and regional nodal radiation therapy, she developed a marked soft tissue reaction to radiation. She underwent over 10 right chest wall open wound radical debridements resulting in a tissue defect of 25 cm in length, by 20 cm in width, by 10 cm in depth. Despite surgical debridement, intravenous antibiotics, hyperbaric oxygen therapy, colistin spray therapy, and heat lamp therapy, the infection failed to resolve and the wound failed to heal. She was left with an open wound that was extremely painful and required chronic pain management with opioids. The patient later was found to have developed a multidrug-resistant Pseudomonas infection in her wound. However, the experimental placement of a porcine bladder matrix (ACell©, Inc., Columbia, MD) on the wound resulted in the complete relief of pain just three days after the application of the product. After two weekly applications of ACell©, her infection completely resolved and she was beginning to grow islands of new epidermis over her non-healing mastectomy wound.

## Introduction

Postmastectomy infections can be a debilitating complication to the patient and pose a challenge to the surgeon left to manage such infections. Infection following mastectomy with same day breast implant reconstruction is not an uncommon entity. Additional risk factors such as radiation therapy, diabetes mellitus, and tobacco abuse increase the risk of such infections. *Pseudomonas aeruginosa* infection has ranked among the top causes of these bacterial infections in a number of studies [1]. The empiric treatment of such infections is broad-spectrum antibiotics [2]. If the infection progresses to ulcerative lesions of ecthyma gangrenosum, then surgical treatment should also be performed in addition to a course of antibiotic therapy [3]. 

Some infections can become resistant to typical treatments, especially when colonized by a multidrug resistant, *Pseudomonas aeruginosa.* The surgeon is left to identify alternate modalities in an effort to heal the wound and allow the patient to recover. In this case report, we present one successful attempt at the treatment of a difficult to manage postmastectomy wound. We found success in managing a difficult wound with a new product urinary bladder matrix (UBM), which is available in a sheet form (Cytal® Wound Matrix, ACell, Inc., Columbia, MD) and powder form (MicroMatrix®, ACell, Inc., Columbia, MD). UBM is an acellular extracellular matrix graft derived from the inner lining of a porcine urinary bladder that has cleared indications for use in wound management and surgical reinforcement of soft tissue. It contains an epithelial basement membrane and acts as a scaffold that facilitates the repair and remodeling of damaged tissue [4]. The main use of UBM is the management of wounds, such as surgical wounds, diabetic ulcers, pressure ulcers, and burns, but many cases have shown its usefulness in other clinical indications [5]. In this case report, we show how UBM facilitated healing of a recalcitrant mastectomy wound refractory to all other modalities of the treatment. Informed consent statement was obtained for this study.

## Case presentation

We present the case of a 52-year-old female with morbid obesity (body mass index of 40.8), noninsulin dependent diabetes mellitus and tobacco abuse who presented with Stage IIIA (T3, N2, Mo) infiltrating ductal carcinoma. The patient received neoadjuvant chemotherapy prior to bilateral skin-sparing total mastectomies with right axillary sentinel lymphadenectomy and staged reconstruction with temporary breast implants. Two months after chest wall and regional nodal radiation therapy, she developed a marked soft tissue reaction to radiation. She underwent a radical debridement of her right chest wall open wound resulting in a tissue defect of 25 cm in length, by 20 cm in width and by 10 cm in depth.

**Figure 1:**
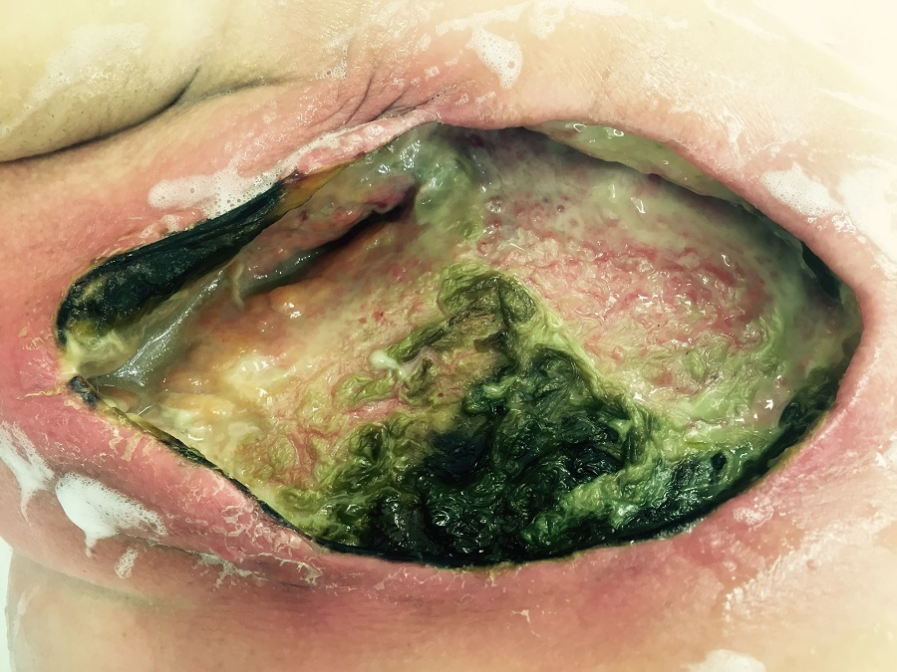
Picture of right breast mastectomy wound Wound that was found to be infected with multidrug resistant *Pseudomonas aeruginosa* refractory to over ten radical surgical debridements, intravenous broad spectrum antibiotics, colistin topical spray therapy, heat lamps, and hyperbaric oxygen therapy prior to placement of porcine bladder matrix

Despite two rounds of surgical debridement, intravenous antibiotics, hyperbaric oxygen therapy, one year of wound care by a wound care specialists, the infection failed to resolve and wound failed to heal leaving her with a large, open defect. She required chronic medication for her pain management. The patient later was found to have developed a multidrug-resistant Pseudomonas wound infection  (Figure 1).

**Figure 2:**
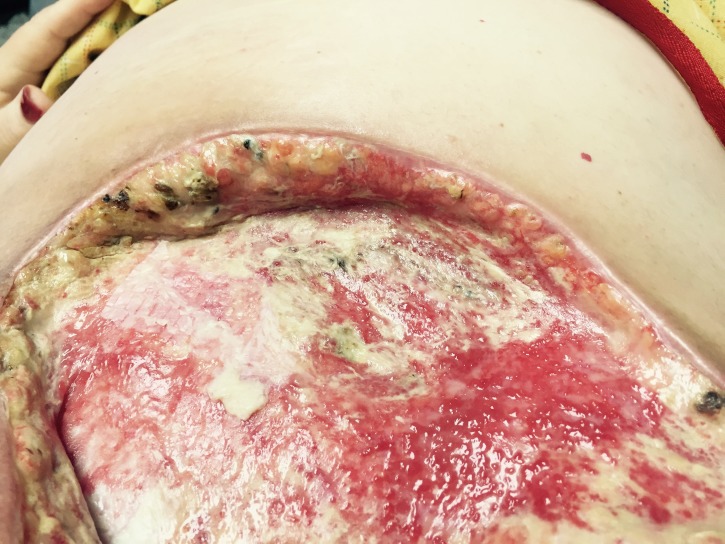
Three days after implantation of porcine bladder matrix. Signs of *Pseudomonas aeruginosa* infection clinically decreasing, healthy granulation tissue visible

Lack of other treatment modalities led the surgeon to experimentally place a product composed of a porcine urinary bladder matrix. Three days after the application of the product, the patient reported that her pain was now manageable without opioid medication (Figure 2). Her wound care regiment consistent of weekly painless applications of UBM powder matrix and sheet epithelial basement membrane on top of powder on the wound. The wound was then covered with lubricating jelly and vaseline gauze. On top of the vaseline gauze, plain gauze was placed and the wound was secured with tape. The patient then wore the same dressing without changing it for days. The patient was instructed to come back for dressing every week to the clinic.

**Figure 3:**
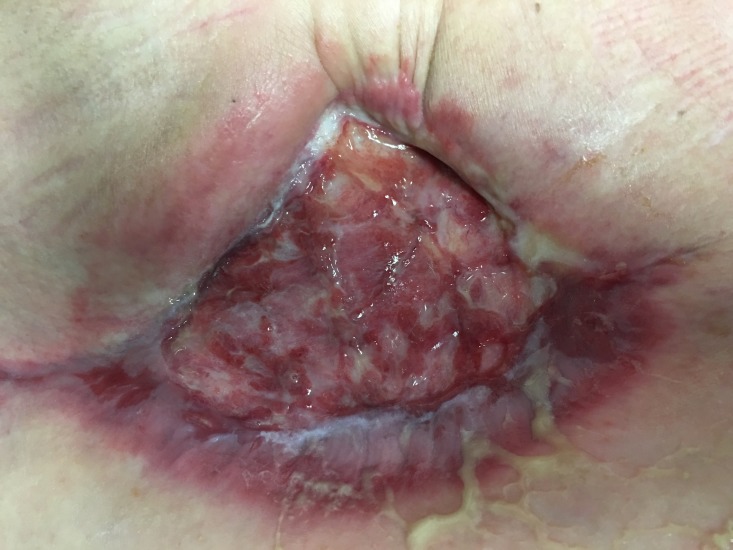
Approximately two months since initial implantation of porcine bladder matrix The wound is contracting and healing by secondary intention. No signs of infection and patient's pain has completely resolved

**Figure 4:**
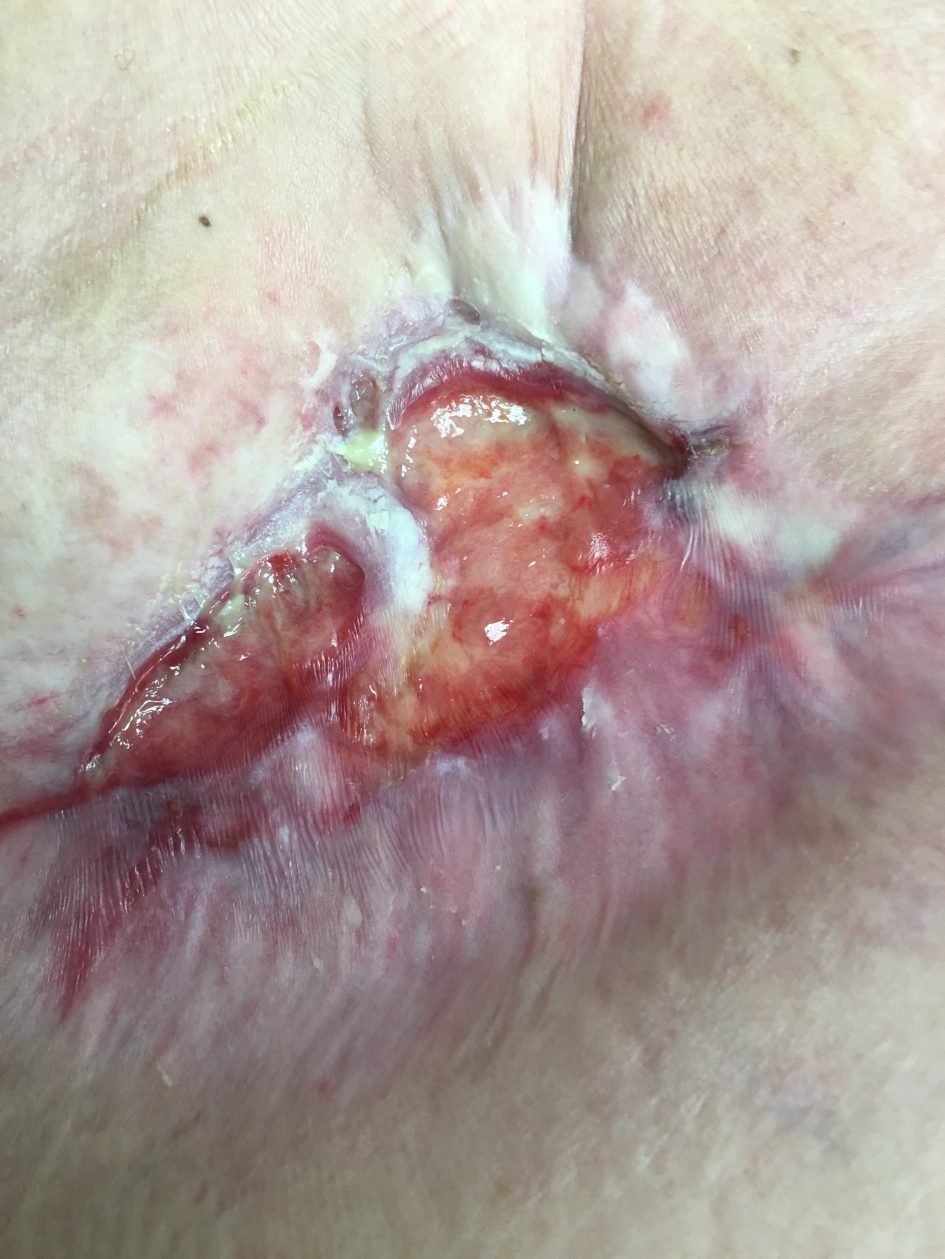
Island of epithelium is seen growing across the wound after approximately three months of weekly treatments of urinary bladder matrix (UBM) placements

**Figure 5:**
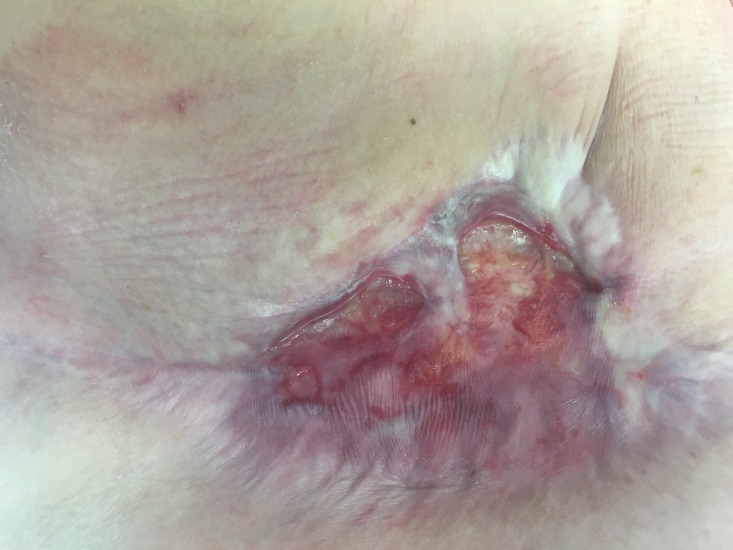
Latest picture of mastectomy defect Six months out from initial porcine bladder matrix placement

After two weekly applications, her infection had completely resolved and she was beginning to grow islands of new epidermis over her chronically open mastectomy wound (Figure 4). By three months, her wound had begun contracting. Four months after placement of UBM, her wound has decreased in size to approximately 3 cm in length by 2 cm in depth and 2 cm in width (Figure 3). Her pain has completely resolved, and the wound is growing epithelial islands which will eventually cover the entirety of the granulation tissue that is in her wound (Figure 5).

## Discussion

Numerous techniques such as hyperbaric oxygen therapy and negative-pressure wound therapy have been used to promote the closure of chronic wounds in previously radiated tissue with mixed success. Hyperbaric oxygen treatment improves oxygenation and neovascularization as well as decreases the inflammation associated with chronic wounds [6]. Infection of chronically nonhealing wound poses an even greater challenge. The relationship between chronically nonhealing wounds and infection is synergistic.

Chronic wounds are more susceptible to infection because they are more likely to be exposed to pathogenic bacteria and an infection will prevent the wound from healing properly which only further increases a person’s susceptibility. Extracellular matrix (ECM) biomaterials, including urinary bladder matrix (UBM), have been available for the past decade and have been shown to stimulate wound closure in animal models and clinical studies by facilitating granulation, epithelialization, and cell growth [7]. The mechanism by which UBM facilitates a more robust healing response is not fully understood but is thought to be at least in part due to an immune response that is characterized by alternatively activated macrophages and T helper cells [8- 9]. It is thought that the modulated immune response facilitates robust angiogenesis, the participation of progenitor cells and resistance to infection.

There is a paucity data that demonstrates utilization of ECM biomaterials in the treatment of chronically infected irradiated wounds. There is only one case series that describes successful healing in chronic wounds in previously irradiated tissue after application of UBM [10]. That case series, coupled with the current report of successful management of a difficult wound with UBM after all other modalities of treatment had failed suggests that further well-controlled clinical studies are warranted. Studying the use of this protocol with other chronic, infected wounds could lead to the development of new standards of treatments that would greatly decrease wound complications and hasten the healing process.

## Conclusions

In this case report, we present a patient with an irradiated, chronically infected multidrug-resistant (MDR) Pseudomonas infection that failed to respond to typical wound care modalities. The placement of a porcine urinary bladder matrix on a wound that had persisted for one year resulted in the complete alleviation of pain just three days after the application of the product. After two weekly applications of UBM, the wound infection completely resolved and she was beginning to grow islands of new epidermis over her nonhealing mastectomy wound. Further research is warranted on the use of UBM devices for the management of infected, difficult to treat wounds.

## References

[1] Vilar-Compte D, Jacquemin B, Robles-Vidal C (2004). Surgical site infections in breast surgery: The case-control study. World J Surg.

[2] Justin CB, Cathy C, Marissa T (2015). Breast implant–associated Infections: The role of the national surgical quality improvement program and the local microbiome. Plast Reconstr Surg.

[3] Seront E, Kidd F, Metz T (2016). Atypical case of ecthyma gangrenosum mimicking a breast cancer recurrence. BMJ Case Rep.

[4] Chun Carol LY, Chhabra N, Houser SM (2016). Novel treatment of a septal ulceration using an extracellular matrix scaffold (septal ulceration treatment using ECM). Am J Otolaryngol.

[5] Justine KS, Alexander JK, Blair SJ (2016). New innovations for deep partial-thickness burn treatment with ACell MatriStem Matrix. Adv Skin Wound Care.

[6] Chiang IH, Chang SC, Wang CH (2017). Management of necrotising fasciitis secondary to abdominal liposuction using a combination of surgery, hyperbaric oxygen and negative pressure wound therapy in a patient with burn scars. Int Wound.

[7] Neill TJ, Badylak SF (2015). The use of biologic scaffolds in the treatment of chronic nonhealing wounds. Adv Wound Care.

[8] Sadtler K, Sommerfeld SD, Wolf MT (2017). Proteomic composition and immunomodulatory properties of urinary bladder matrix scaffolds in homeostasis and injury. Semin Immunol.

[9] Brown BN, Londono R, Tottey S (2012). Macrophage phenotype as a predictor of constructive remodeling following the implantation of biologically derived surgical mesh materials. Acta Biomaterialia.

[10] Rommer EA, Peric MBA, Wong A (2013). Urinary bladder matrix for the treatment of recalcitrant nonhealing radiation wounds. Adv Skin Wound Care.

